# ﻿*Sipadantoniusroihani* gen. et sp. nov., a new genus and species of Pseudocyclopidae Giesbrecht, 1893 (Copepoda, Calanoida) from the marine cave “Turtle Tomb” of Sipadan Island, Sabah, Malaysia

**DOI:** 10.3897/zookeys.1219.133132

**Published:** 2024-12-06

**Authors:** Chaichat Boonyanusith, Koraon Wongkamhaeng, Abdul-Rahim Azman

**Affiliations:** 1 School of Biology, Faculty of Science and Technology, Nakhon Ratchasima Rajabhat University, Nakhon Ratchasima 30000, Thailand Nakhon Ratchasima Rajabhat University Nakhon Ratchasima Thailand; 2 Department of Zoology, Faculty of Science, Kasetsart University, Bangkok 10900, Thailand Kasetsart University Bangkok Thailand; 3 Department of Earth Sciences and Environment, Faculty of Science and Technology, Universiti Kebangsaan Malaysia, 43600 UKM Bangi, Selangor, Malaysia Universiti Kebangsaan Malaysia Bangi Malaysia

**Keywords:** Crustacea, Southeast Asia, systematics, taxonomy, Zooplankton

## Abstract

A new genus and species of the family Pseudocyclopidae, *Sipadantoniusroihani***gen. et sp. nov.**, was described based on specimens collected using a light trap in the marine cave of Sipadan Island, Sabah, Malaysia. The new genus is most related to *Pinkertonius*, primarily based on the similarity observed in the armament of ancestral segment IV of the male antennules, the armament of the female P5 Exp-3, the segmentation of the male P5, the armament of the maxillular basal exite, and the relative length of the ancestral segment XXVII of the antennules. Nevertheless, it distinguishes itself from *Pinkertonius* and all other genera of the family by the absence of the lateral seta of the basis of all swimming legs, the presence of an inner seta on the coxa of the female P5, the reduction of furcal setae I and III, as well as the specific armament of the ancestral segment XX of the antennules and the maxillular coxal endite. The female of *Sipadantoniusroihani***gen. et sp. nov.** has aesthetascs on the ancestral segments IV and XX of the antennules, as well as six setae on the maxillular coxal endite, exhibiting the most plesiomorphic characteristics of the family Pseudocyclopidae. The latter characteristic has not been recorded in the order Calanoida. It was hypothesised that the new species was a particle feeder living in the pelagic zone of the marine cave. The existence of the new species supported the assumption that the regional distribution of the family Pseudocyclopidae exhibited the Tethyan track, which might have been the subsequent result of the colonisation of the habitats prior to the closure of the Tethys Sea.

## ﻿Introduction

Based on the phylogenetic research conducted by Bradford-Grieve et al. (2014), 14 calanoid genera were recognised as members of the family Pseudocyclopidae Giesbrecht, 1893, which included *Badijella* Kršinic, 2005, *Boholina* Fosshagen, in Fosshagen & Iliffe, 1989, *Brattstromia* Fosshagen, in Fosshagen & Iliffe, 1991, *Exumella* Fosshagen, 1970, *Exumellina* Fosshagen, 1998, *Hondurella* Suárez-Morales & Iliffe, 2007, *Normancavia* Fosshagen & Iliffe, 2003, *Pinkertonius* Bradford-Grieve, Boxshall & Blanco-Bercial, 2014, *Placocalanus* Fosshagen, 1970, *Pseudocyclops* Brady, 1872, *Ridgewayia* Thompson I.C. & Scott A., 1903, *Robpalmeria* Fosshagen & Iliffe, 2003, *Stargatia* Fosshagen & Iliffe, 2003 and *Stygoridgewayia* Tang, Barron & Goater, 2008. The family was united with the family Epacteriscidae Fosshagen, 1973, thereby forming the superfamily Pseudocyclopoidea Giesbrecht, 1893, which was the basal clade of the order Calanoida Sars, 1903, based on molecular phylogenetic study (Bradford-Grieve et al., 2014). Among the listed genera, *Pinkertonius* was regarded as the basal taxon of the family (Bradford-Grieve et al. 2014).

Pseudocyclopid copepods are prevalent in shallow benthopelagic or anchialine cave habitats within tropical and subtropical marine waters globally, as documented by various researchers (e.g., Fosshagen and Iliffe 1989, 1991, 1998, 2003; Boxshall and Jaume 2012; Bradford-Grieve et al. 2014; Ohtsuka et al. 1996, 2000; Suárez-Morales and Iliffe 2007; Figueroa 2011; Moon and Soh 2014). Additionally, some genera and species are recognised from freshwater subterranean habitats, including *Stygoridgewayiatrispinisa* Tang, Barron & Goater, 2008, *Boholinalaorsriae* Boonyanusith, Wongkamhaeng & Athibai, 2020 and *Boholinareducta* Tran & Chang, 2020 (Tang et al. 2008; Boonyanusith et al. 2020; Tran and Chang 2020).

In the past decade, a great deal of research was conducted regarding the zooplankton ecosystem of Malaysian waters (Rezai et al. 2009; Yoshida et al. 2012; Nakajima et al. 2013; Balqiah and Rahim 2021), resulting in the documentation of more than 230 species. Nevertheless, the documentation regarding the prevalence of endemic zooplankton species remained insufficient. To date, only four species were recognised as endemic or host-specific, namely *Brachiellamalayensis* Ohtsuka, Piasecki, Ismail & Kamarudin, 2020, *Kensakiaparva* Harris V.A. & Iwasaki, 1997, *Labidocerajaafari* Othman, 1986 and *Sipadaniacelerinae* Humes & Lane, 1993. A recent biological survey conducted at the Sipadan Turtle Tomb yielded a significant number of specimens of calanoid copepods, which could not be assigned to any described species.

Morphologically, the new species appeared to resemble the genus *Pinkertonius* in the armament of the ancestral segment IV of the male antennule, the armament of the female P5 Exp-3, and the segmentation of both rami of the male P5. However, it bore a specific combination of the characteristics, particularly regarding the armament of the swimming legs and mouthparts; therefore, the status of a new genus was justified for the Malaysian specimens, and the name of *Sipadantonius* gen. nov. was proposed. The description and illustrations of the new taxon were provided hereafter.

## ﻿Materials and methods

**Figure 1.**
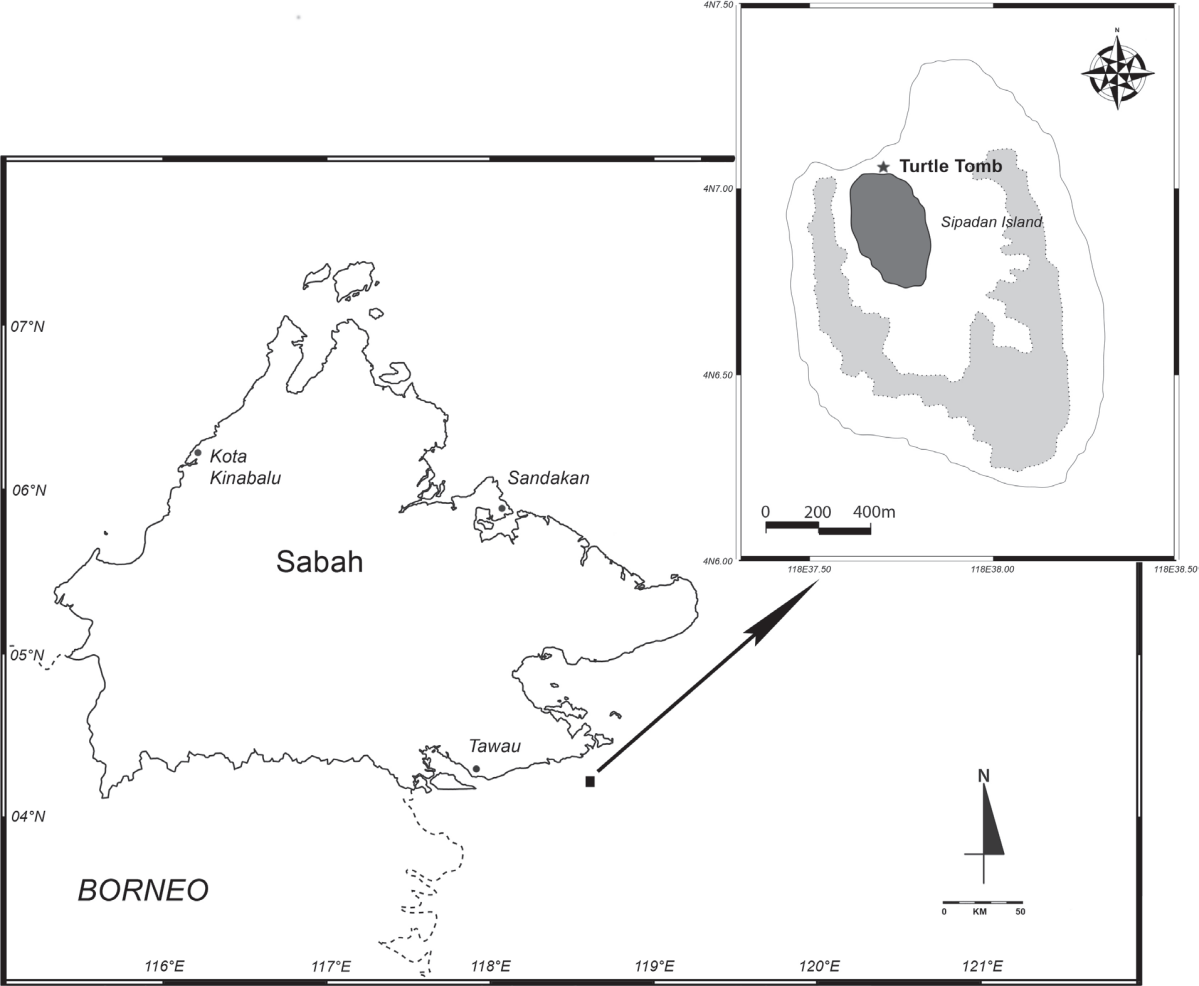
Geographical location of the type locality of *Sipadantoniusroihani* gen. et sp. nov.

Specimens were collected in the Turtle Tomb of Sipadan Island, Sabah, Malaysia, on 3 August 2023 (Fig. 1). Sampling was conducted utilising a modified light trap at a depth of 20–24 m, which was deployed at the bottom substrate for ~ 24 hours. The specimens utilised for morphological descriptions were preserved in 10% buffered formalin prior to examination. Copepods were meticulously sorted under a stereomicroscope, followed by fixation and storage in 70% ethanol in the laboratory. Subsequently, a few specimens were soaked in the mixture of glycerol and 70% ethanol (approximate ratio 1/10 v/v) for 30 min. After that, animals of both sexes were individually transferred into a drop of 40% glycerol on a glass slide and dissected under a stereomicroscope. The material was ultimately mounted with a coverslip and subsequently examined under a Nikon ECLIPSE E200 compound light microscope at 1000 × magnification.

The habitus and appendages were examined and subsequently drawn utilising a drawing tube attached to a compound microscope at 400 × and 1000 × magnifications, respectively. Description was made by adopting the terminology established by Huys and Boxshall (1991) and employing the following abbreviations: **ae** = aesthetasc, **I** = spine, **Endp** = endopod, **Exp** = exopod, **Endp-1 (2, 3)** = proximal (middle, distal) segment of the endopod of the swimming legs, **Exp-1 (2, 3)** = proximal (middle, distal) segment of the exopod of the swimming legs, **P1–P6** = first to sixth swimming legs, **Seta I–VII** = first to seventh furcal seta, **Seta I** = anterolateral accessory seta, **Seta II** = anterolateral seta, **Seta III** = postereolateral seta, **Seta IV** = outer terminal seta, **Seta V** = inner terminal seta, **Seta VI** = terminal accessory seta, and **Seta VII** = dorsal seta.

### ﻿Specimen repositories

The type material was deposited at the Universiti Kebangsaan Malaysia Muzium Zoologi (UKMMZ), Malaysia and the Sabah Parks Zoological Collection in Semporna, in accordance with the requirements stated in the Sabah Biodiversity Council – License for Transfer Ref. No. JKM/MBS.1000-2/3 JLD.5 [41].

## ﻿Taxonomic account

### ﻿Order Calanoida Sars, 1903

**Superfamily Pseudocyclopoidea Giesbrecht, 1893**

**Family Pseudocyclopidae Giesbrecht, 1893**

#### ﻿Genus Sipadantonius gen. nov.

Taxon classificationAnimaliaCalanoidaPseudocyclopidae

B73E5302-54E8-51D1-A7AE-DF7DE44A0CC8

https://zoobank.org/EF5F740F-CEAE-4CC4-BD2E-4E4FE621C904

##### Diagnosis.

**Female**: Elliptical body. Single plate rostrum, with rounded tip, lacking rostral filaments. Six-segmented prosome. Symmetrical and rounded postero-lateral corners of the fifth pedigerous somite. Four-segmented and symmetrical urosome. Double-somite genital with genital operculum ventromedially. Symmetry furcal rami, with serrated hyaline frill on distal margin, armed with five setae on the tip of the ramus, without furcal setae I and III; spiniform seta II; normal-developed seta V, slightly longer than seta IV. Antennule with 26 segments-; ancestral segments II and fused III; ancestral segments X and partly fused XI; relatively short ancestral segment XXVII; with aesthetasc on ancestral segments I, III–XXI, XXV, and XXVII. Antennae with two-segmented Endp and nine-segmented Exp; segments I–VII of Exp each with one seta. Mandibles with un-modified coxal gnathobase, five-segmented Exp and two-segmented Endp; a distal segment of Endp with ten setae. Maxillulae with nine setae on coxal epipodite and six setae on coxal endite; unarmed basal exite; two-segmented, un-modified Endp. Seven-segmented maxillae lack an outer seta on the outer margin of the coxa. Eight-segmented maxillipeds; length of segments II–VI of Endp is equivalent to syncoxa; transformed proximalmost seta on segment III, with feather-like tip. Three-segmented P1–P5 with both rami; coxa with seta on the distomedial corner; the basis of all swimming legs lacking lateral seta, that of P2–P5 with cuticular window representing the remnant of armament on outer margin; the posterior surface of the basis of P1 with the curved hyaline process. Spine and setal complements of Exp-3 of P2–P5: 2.3.3.3 and 5.5.5.4, respectively; setal complement of Endp-3 of P2–P5: 8.7.7.6. Distolateral corner of P1Endp-1 and Endp-2 of P1–P4 with bifid indentures; distolateral corner of both Endp-1 and Endp-2 of P5 un-fid. *Male*: Body shape, rostrum, prosome, mouthparts, furcal rami, and P1–P4 are identical to those of females. Slightly asymmetrical urosome. The left antennule is identical to that of the female; the 22-segmented right antennule, weakly geniculate; fused ancestral segments II–IV, XXI–XXIII, and XXIV–XXV. P5 is asymmetrical, with both three-segmented rami; basis with cuticular pores representing the remnant of armament on outer margin; Endp-1 of left and right legs lacks inner seta; separated Exp-2 and Exp-3; those of the right leg are modified to function as grasping organ.

##### Type species.

*Sipadantoniusroihani* sp. nov.

##### Etymology.

Named after the type locality, Sipadan Island, Sabah, Malaysia, in combination with the -*tonius* stem from the existing generic name *Pinkertonius* Bradford-Grieve, Boxshall & Blanco-Bercial, 2014, alluding to the similarity of the genus *Pinkertonius*. The gender is masculine.

#### ﻿ Sipadantonius roihani sp. nov.

Taxon classificationAnimaliaCalanoidaPseudocyclopidae

80A41A6A-38FB-5E21-84ED-1769FD526F7A

https://zoobank.org/C38B34EF-2C23-4A8D-9396-616EAD33D82D

**Figure 2.**
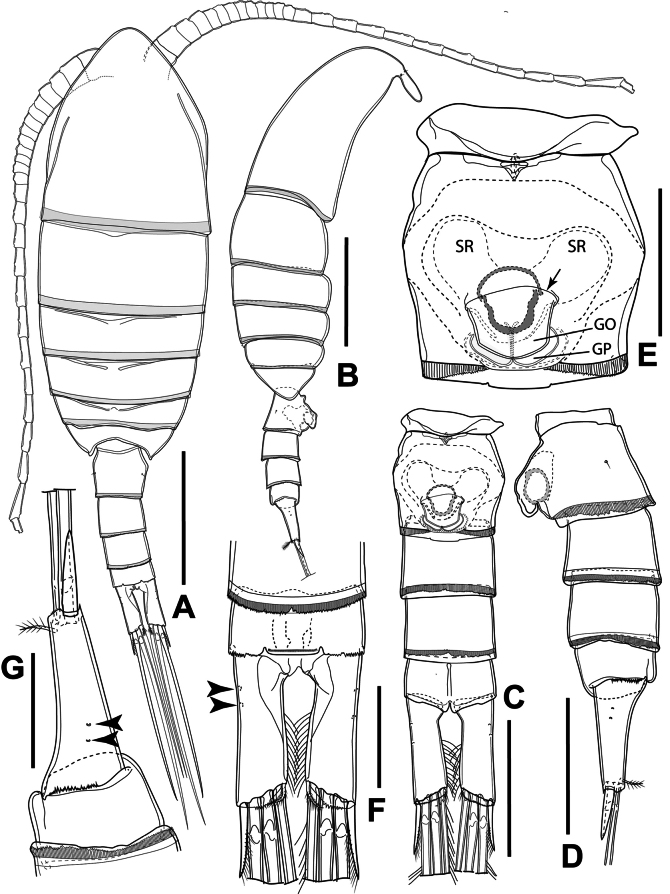
*Sipadantoniusroihani* gen. et sp. nov. female: **A** habitus, dorsal view **B** habitus, lateral view **C** urosome, ventral view **D** urosome, lateral view **E** genital double-somite, ventral view **F** furcal rami, dorsal view **G** furcal rami, lateral view. Arrowheads indicate integumental pores. Scale bars: 200 μm (**A, B**); 100 μm (**C, D**); 50 μm (**E−G**).

**Figure 3.**
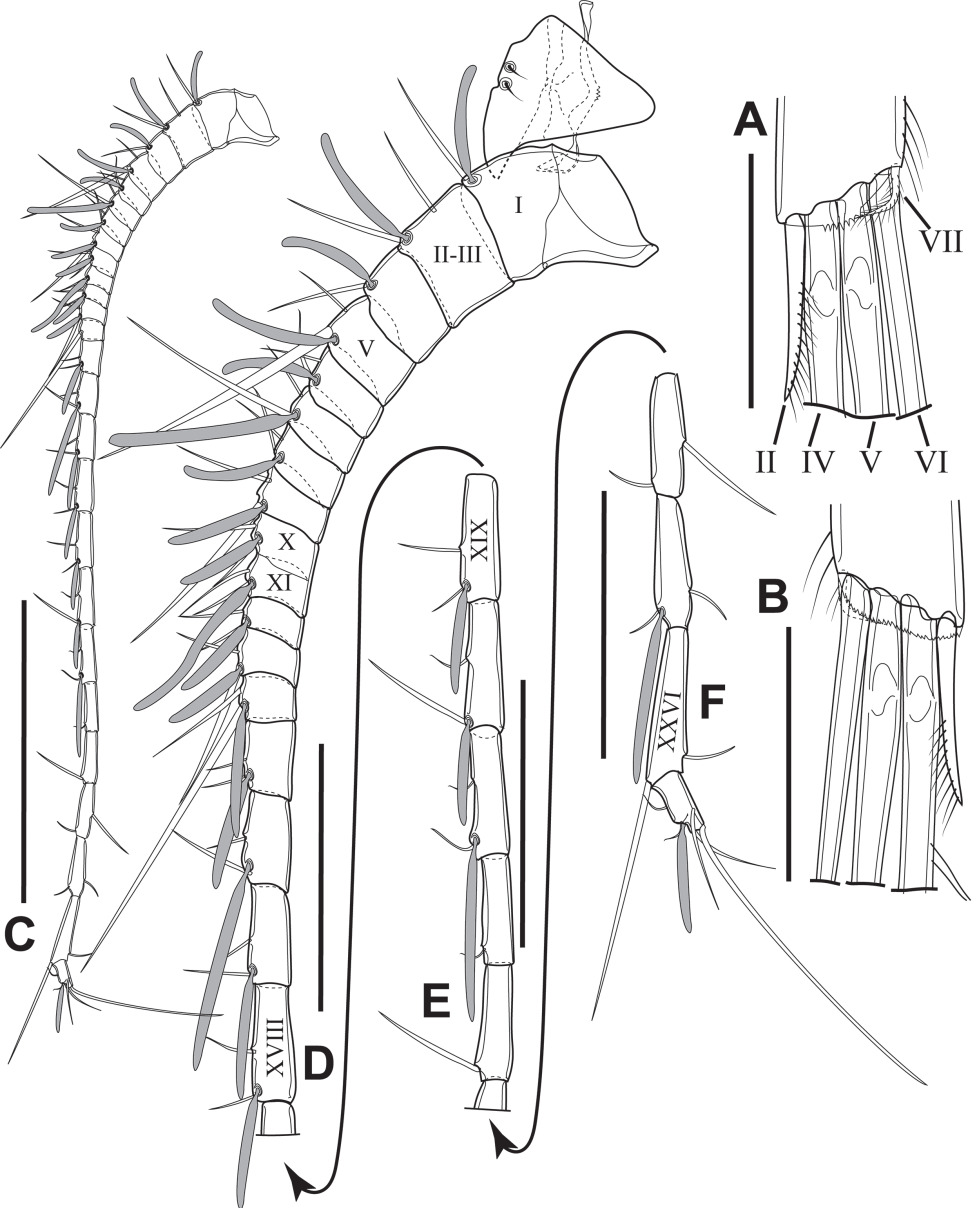
*Sipadantoniusroihani* gen. et sp. nov. female: **A** tip of furcal ramus, dorsal view **B** tip of furcal ramus, ventral view **C** antennule **D** rostrum and segments 1–17 of antennule **E** segments 18–22 of antennule **F** segments 23–26 of antennule. Roman numerals on antennule correspond to ancestral segments. Scale bars: 50 μm (**A, B**); 200 μm (**C**); 100 μm (**D−F**).

**Figure 4.**
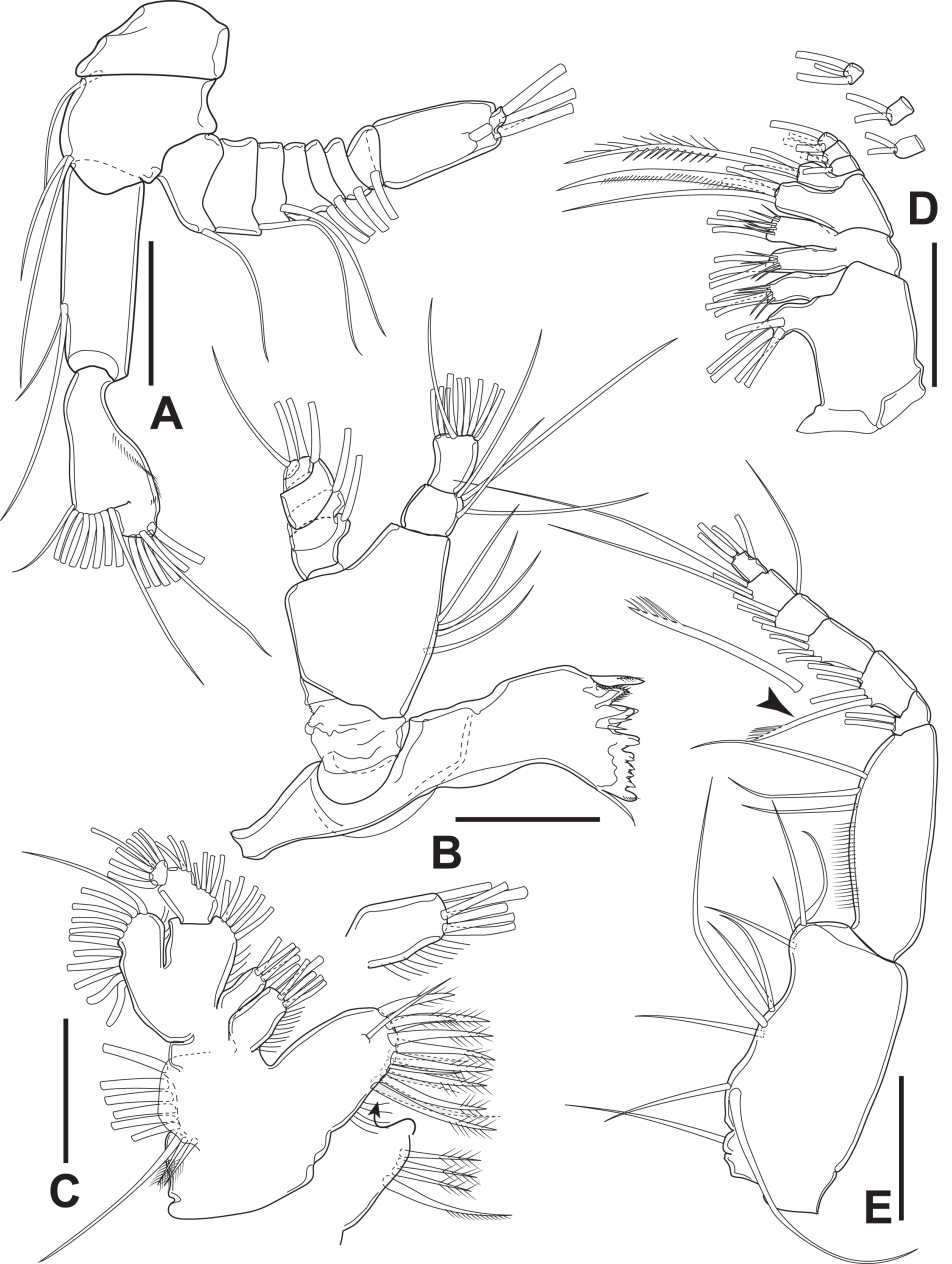
*Sipadantoniusroihani* gen. et sp. nov. female: **A** antenna **B** mandible **C** maxillule, with picture of setae on posterior surface of praecoxal arthrite and enlarged picture of coxal endite **D** maxilla **E** maxilliped, with enlarged picture of transformed seta. Arrowhead indicates transformed seta on maxilliped. Scale bars: 50 μm.

**Figure 5.**
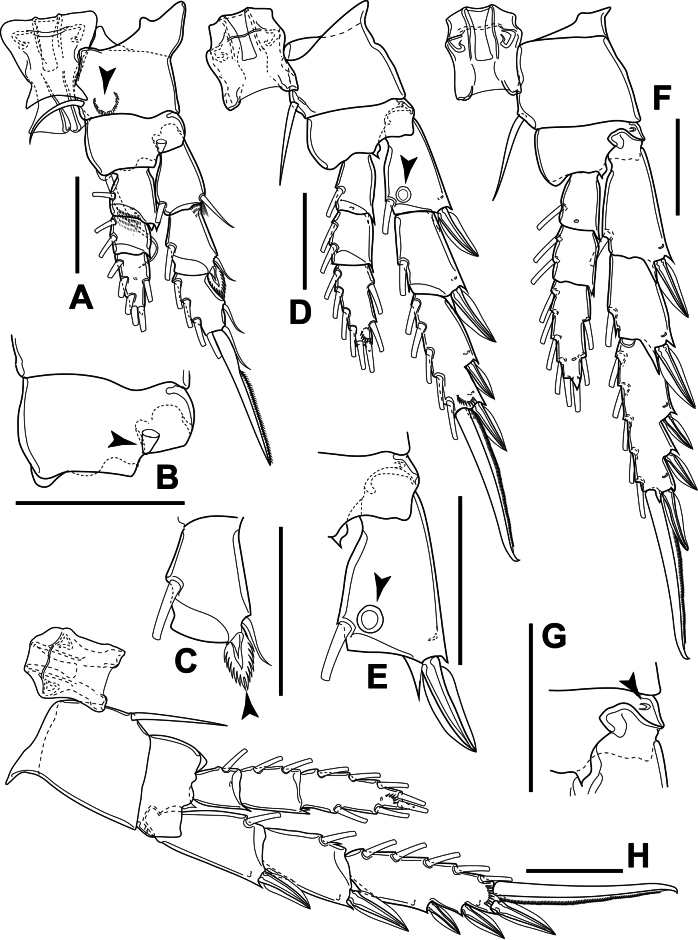
*Sipadantoniusroihani* gen. et sp. nov. female: **A**P1**B** basis of P1, posterior surface **C**P1Exp-2 **D** P2 **E** P2 Exp-1, posterior surface **F** P3 **G** lateral margin of basis of P3 **H** P4. Arrowheads indicate the important characteristic of the structure. Scale bars: 50 μm.

**Figure 6.**
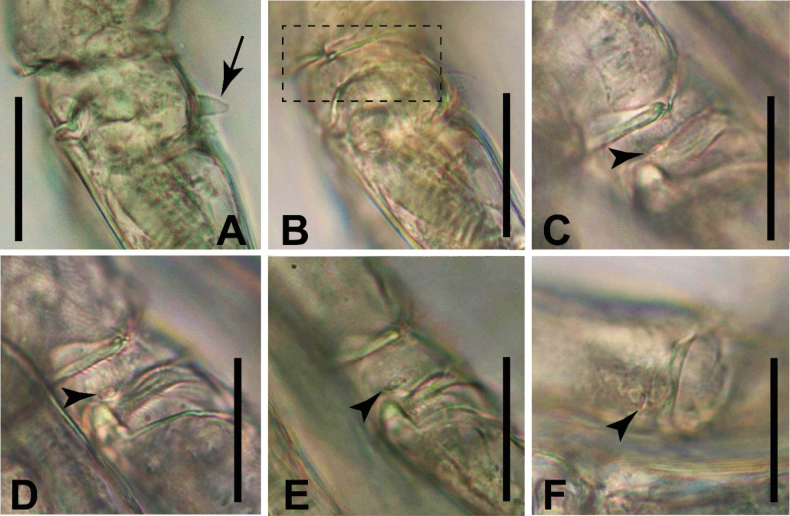
*Sipadantoniusroihani* gen. et sp. nov. photographs of lateral surface of basis of swimming legs, female (**A−E**) and male (**F**): **A** posterior hyaline process on basis of P1 (indicated by arrow) **B**P1**C** P3 **D** P4 **E, F** P5. Arrowheads indicate cuticular windows on lateral margin of basis. Scale bars: 10 μm.

Figs 2 , 3 , 4 , 5 , 6 (female); 6–8 (male)

##### Material examined.

***Holotype*** • ♀ (adult), 0.95 mm long; 3 August 2023; coll. Azman, B.A.R.; light trap; UKMMZ-1631. ***Allotype*** • ♂ (adult), 0.87 mm long, collection data for holotype; UKMMZ-1632. ***Paratypes*** • 1 ♀ (adult) and 1 ♂ (adult); each was utterly dissected and mounted on a slide in glycerol and then sealed with nail varnish; the data was identical to that of the holotype; UKMMZ-1633–1634.

##### Additional material.

• 2 ♂♂ (adult); the data was identical to that of the holotype; preserved in 70% ethanol, subsequently retained in collection of the Sabah Parks Zoological Collection in Semporna, Sabah.

##### Type locality.

The marine cave “Turtle Tomb”, Sipadan Island, Sabah, Malaysia; the entrance is located at 4°07'04.8"N, 118°37'41.0"E. Samples were collected in the cave at a depth of 22.0 meters below the sea surface, ~ 100 meters from the entrance.

##### Description of adult female.

Body (Fig. 2A, B) with a total length of 0.91–0.95 mm (measured from anterior margin of cephalosome to tip of furcal rami; mean: 0.93 mm; *n* = 3), slightly dorsoventrally flattened; integument covered with hair-like spinules. Prosome six-segmented, elliptical, ~ 70% of body length and 2.2 × as long as urosome, ~ 2.3 × as long as wide, with greatest width at posterior margin of the first pedigerous somite (P1-bearing somite) (Fig. 2A). Cephalosome and all pedigerous somites free, with smooth hyaline frill on posterior margin of cephalosome and first four pedigerous somites (Fig. 2A, B); postero-lateral corners symmetrical and rounded (Fig. 2B). Naupliar eye not discernible. Urosome four-segmented, comprising genital double-somite, and three free abdominal somites; all somites with finely serrated hyaline frill on posterior margin (Fig. 2C–F). Genital double-somite barrel-shaped (Fig. 2E), ~ 27% of urosome length, as long as wide, with greatest width at mid-length of double-somite, with lingual-shaped genital operculum ventromedially; two gonoporal plates triangular-shaped, partially hidden under genital operculum (Fig. 2E). Three free abdominal somites subequal in length. Anal somite with slightly developed anal operculum; posterior margin with serrated hyaline frill dorso-laterally (Fig. 2F, G); anal operculum with smooth free margin, ornamented with row of spinules (Fig. 2F).

Furcal rami (Fig. 2F, G) symmetrical, parallel, each ~ 3 × as long as wide, with serrated hyaline frill on distal margin and two cuticular pores laterally (Fig. 2F, G); furcal setae I and III absent; furcal seta II spiniform, with setulae along inner margin; seta IV shorter than seta V, with breaking planes and plumose; seta V longest, with breaking plane and plumose, sub-equal to urosome length, ~ 1.2 × as long as seta IV; seta VI plumose, ~ 0.8 × as long as seta V; seta VII very short and plumose, inserted beside seta VI (Fig. 3A). Length ratio of furcal setae II to ramus length ~ 0.5 and the ratio of setae to ramus length from seta IV to seta VII: 3.3: 3.9: 3.0: 0.2.

Rostrum (Figs 2B, 3D) well developed, single plate and V-shaped; base broad, completely fused to anterior margin of cephalic shield and tapering to rounded tip between bases of antennules, with two sensillae, lacking rostral filaments.

Antennules (Figs 2A, 3C, D) symmetrical, representing 26-segmented, reaching distal margin of urosomite 2; ancestral segments II and III completely fused, representing evident segment II; ancestral segments X–XI partly fused, with remnant of ancestral articulation; ultimate segments ~ 1/3 of the length of pre-ultimate segment. Armature formula as follows (Roman numerals correspond to ancestral segment): 1+ae (I), 2+ae (II–III), 1+ae (IV), 2+ae (V), 2+ae (VI), 2+ae (VII), 2+ae (VIII), 2+ae (IX), 2+ae (X), 2+ae (XI), 2+ae (XII), 2+ae (XIII), 2+ae (XIV), 2+ae (XV), 2+ae (XVI), 2+ae (XVII), 2+ae (XVIII), 2+ae (XIX), 2+ae (XX), 2+ae (XXI), 1 (XXII), 1 (XXIII), 2 (XXIV), 2+ae (XXV), 2 (XXVI), 5+ae (XXVII–XXVIII).

Antennae (Fig. 4A) biramous. Coxa short, bearing one seta on distomedial corner. Basis with two setae on distomedial corner. Exp nine-segmented, setal formula from proximal to distal segments: 1.1.1.1.1.1.1.1.3. Endp two-segmented; proximal segment bearing two setae inserted at the same place on medial margin; distal segment bilobed, bearing nine apical setae on medial lobe and seven apical setae on distal lobe, with curved row of spinules on outer margin.

Mandibles (Fig. 4B) with sclerotised gnathobase comprising nine cuspidate teeth and one small dorsal seta on cutting edge. Mandibular palp biramous; basis with four setae on inner margin. Exp five-segmented, setal formula from proximal to distal segments: 1.1.1.1.2. Endp two-segmented; proximal segment with four setae on distomedial corner; distal segment short, with ten apical setae.

Maxillulae (Fig. 4C) with praecoxal arthrite bearing nine spinulose and spiniform marginal setae and one smooth marginal seta, with one seta on anterior surface and four setae on posterior surface; proximalmost one on posterior surface longest, unipinnate. Coxal epipodite with nine apical setae; two proximal ones shortest; coxal endite with six apical setae. Basis fused to Endp, proximal and distal endites armed with four and five apical setae, respectively; basal exite knob-like appearance, unarmed. Exp with eleven setae along apical and outer margin. Endp two-segmented, proximal and middle segments completely fused, setal formula: 4.3.7.

Maxillae (Fig. 4D) seven-segmented, comprising praecoxa, coxa, basis and four-segmented Endp. Proximal and distal praecoxal endites with five and three apical setae, respectively. Coxa with two endites, each armed with three apical setae, with long spinules near distal margin of endite. Basis with large basal endite, armed with four apical setae; one of which strong and spiniform. Endp with setal formula: 3.2.2.3.

Maxillipeds (Fig. 4E) eight-segmented, comprising syncoxa, basis, and six-segmented Endp. Syncoxa with four syncoxal endites, setal formula: 1.2.4.4; one seta on second and third endite ~ 2 × as long as seta arising nearby. Basis with three medial setae, with row of spinules on anterior surface. Endp with setal formula: 2.4.4.3.3+1.4; seta 4 on segment II of Endp spiniform, with feather-like tip (Fig. 4G).

P1–P4 (Figs 5A–H, 6A–D) biramous, comprising coxa, basis, and three-segmented rami. Integument ornamented with numerous spinules and short hairs. Intercoxal sclerite sub-rectangular. Coxa with medial seta on distomedial corner. Basis of P2–P4 with cuticular window representing remnant of armament on outer margin (Figs 5G, 6C, D) but the remnant not discernible in P1 (Fig. 6B). Outer spine of P1Exp setiform; those of Exp of P2–P4 stronger and oar-shaped. Distolateral corner of P1Exp-2 drawn out into mint leaf-like process; those of Exp-1 and Exp-2 of P2–P4 slightly extended, with two acutely protrusions beside spine; inner protrusion larger and longer than outer one. Anterior surfaces of Exp-1 and Exp-2 with cuticular pores near insertion of outer spine; Exp-2 with acutely minute process near distomedial corner in P2–P4 but not discernible in P1; Exp-3 with cuticular pores near insertion of both proximalmost outer spine and outer apical one. Endp of all swimming legs with cuticular pores near distal margin of Endp-1 and at distal ~ 1/4 of Endp-3. Outer and outer apical spines of all swimming legs relatively short, oar-shaped; inner apical spine with serrated cuticular expansion on outer margin. Armature of swimming legs as presented in Table 1. Some other characteristics of P1–P4 as following described.

**Table 1.** Armament of P1–P5 in *Sipadantoniusroihani* gen. et sp. nov., (Legend: outer-inner element; outer-apical-inner element; Arabic numerals indicate number of setae; Roman numerals indicate number of spines).

Swimming leg	Coxa	Basis	Exopod			Endopod		
			1	2	3	1	2	3
P1	0-1	0-1	I-1	1-1	II-I-4	0-1	0-2	1-2-3
P2	0-1	0-0	I-1	I-1	II-I-5	0-1	0-2	2-2-4
P3	0-1	0-0	I-1	I-1	III-I-5	0-1	0-2	2-2-4
P4	0-1	0-0	I-1	I-1	III-I-5	0-1	0-2	2-2-3
P5 (female)	0-1	0-0	I-1	I-1	III-I-4	0-1	0-1	2-2-2
Left leg of P5 (male)	0-0	0-0	I-0	I-0	0-I-I	0-0	0-1	2-2-2
Right leg of P5 (male)	0-0	0-0	I-0	I-0	I-0-I	0-0	0-1	2-2-2

P1 (Figs 5A–C, 6A, B). Coxa trapezoidal; anterior surface with triangular expansion on proximomedial corner and oval integumental window near distomedial corner; integumental window surrounded by curved spinules. Basis bearing medial seta on distomedial corner but lacking either lateral seta or remnant of armament on outer margin; posterior surface with short curved hyaline process near insertion of Exp (Fig. 6B). Anterior surface of Exp-1 with row of long spinules on distolateral corner. Endp-1 with bifid indenture on distolateral corner and long spinules along posterior margin; Endp-2 with an acute indenture on distolateral corner.

P2 (Fig. 5D, E). Coxa rectangular. Posterior surface of Exp-1 with circular integumental window near insertion of inner seta; Exp-3 with two outer spines. Endp-1 with an acute indenture on distolateral corner; that of Exp-2 bifid.

P3 (Figs 5F, G, 6C) as that of P2 but Exp-1 lacking integumental window on posterior surfaces and Exp-3 with three outer spines.

P4 (Figs 5H, 6D) Coxa sub-quadrate. Basis and rami as those of P3.

**Figure 7.**
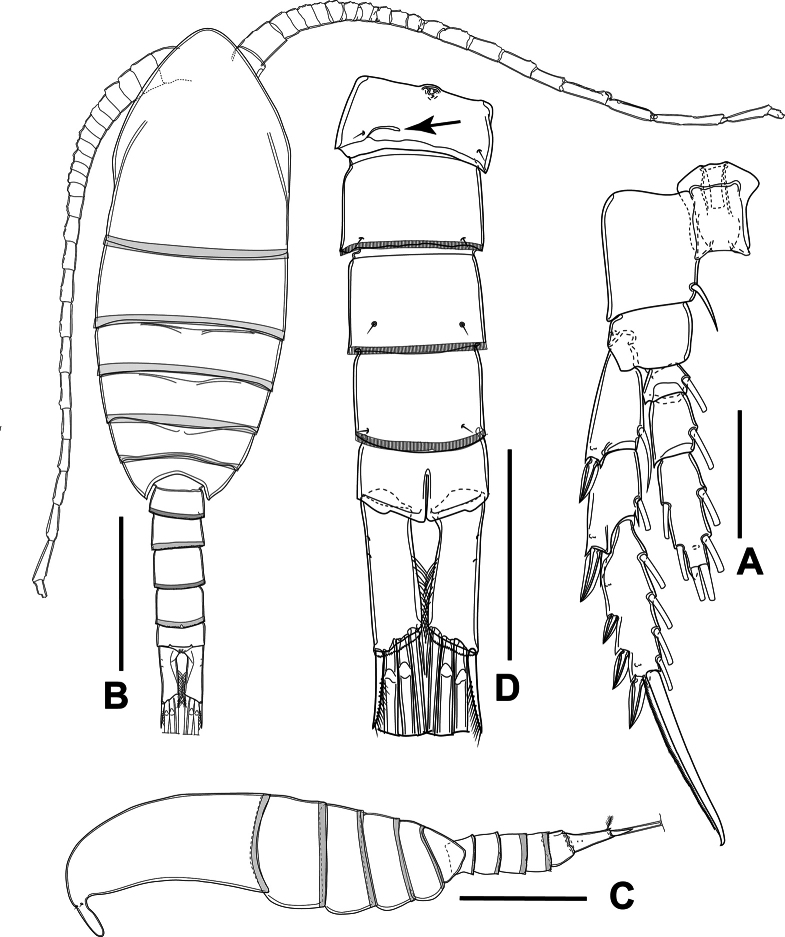
*Sipadantoniusroihani* gen. et sp. nov. female **(A)** and male **(B−D)**: **A** P5 **B** habitus, dorsal view **C** habitus, lateral view **D** urosome, ventral view. Arrow indicates the ventral suture on genital somite. Scale bars: 50 μm (**A, D**); 200 μm (**B, C**).

P5 (Figs 6E, 7A) biramous, with both rami three-segmented; armament as in Table 1. Integument and intercoxal sclerite as described for P2–P4. Coxa rectangular, longer than wide; medial seta shorter than those of P1–P4. Basis, Exp-1 and Exp-2 as in P3 and P4, but inner seta on Exp-1 shorter than those of P1–P4. Exp-3 ~ 2.5 × as long as wide, offset on inner side at distal ~ 1/3 of segment. Endp-1 and Endp-2 with an acute indenture on distolateral corner each; Endp-3 longer than length of Endp-1 and Endp-2 combined. Ornamentation as that of P3 and P4.

##### Description of adult male.

Body (Fig. 7B) with a total length of 0.87 and 0.89 mm (measured from anterior margin of cephalosome to tip of furcal rami; mean: 0.89 mm; *n* = 2). Habitus slightly smaller and slenderer than in female (Fig. 7B, C). Prosome six-segmented, elliptical, ~ 70% of body length and 2.2 × as long as urosome, ~ 2.6 ×as long as wide, with greatest width at posterior margin of the first pedigerous somite. Cephalosome and first three pedigerous somites similar to those in female. Naupliar eye not discernible. Urosome five-segmented; comprising genital somite and four free abdominal somites. Genital somite slightly asymmetrical; ventral surface with gonopore on proximal margin. All free abdominal somites similar in length, each with finely serrated hyaline frill on posterior margin. Anal somite identical to that of female (Fig. 7B, D).

Furcal rami (Fig. 7D) identical to that of female, ~ 3 ×as long as wide. Armament and ornamentation identical to that of female.

**Figure 8.**
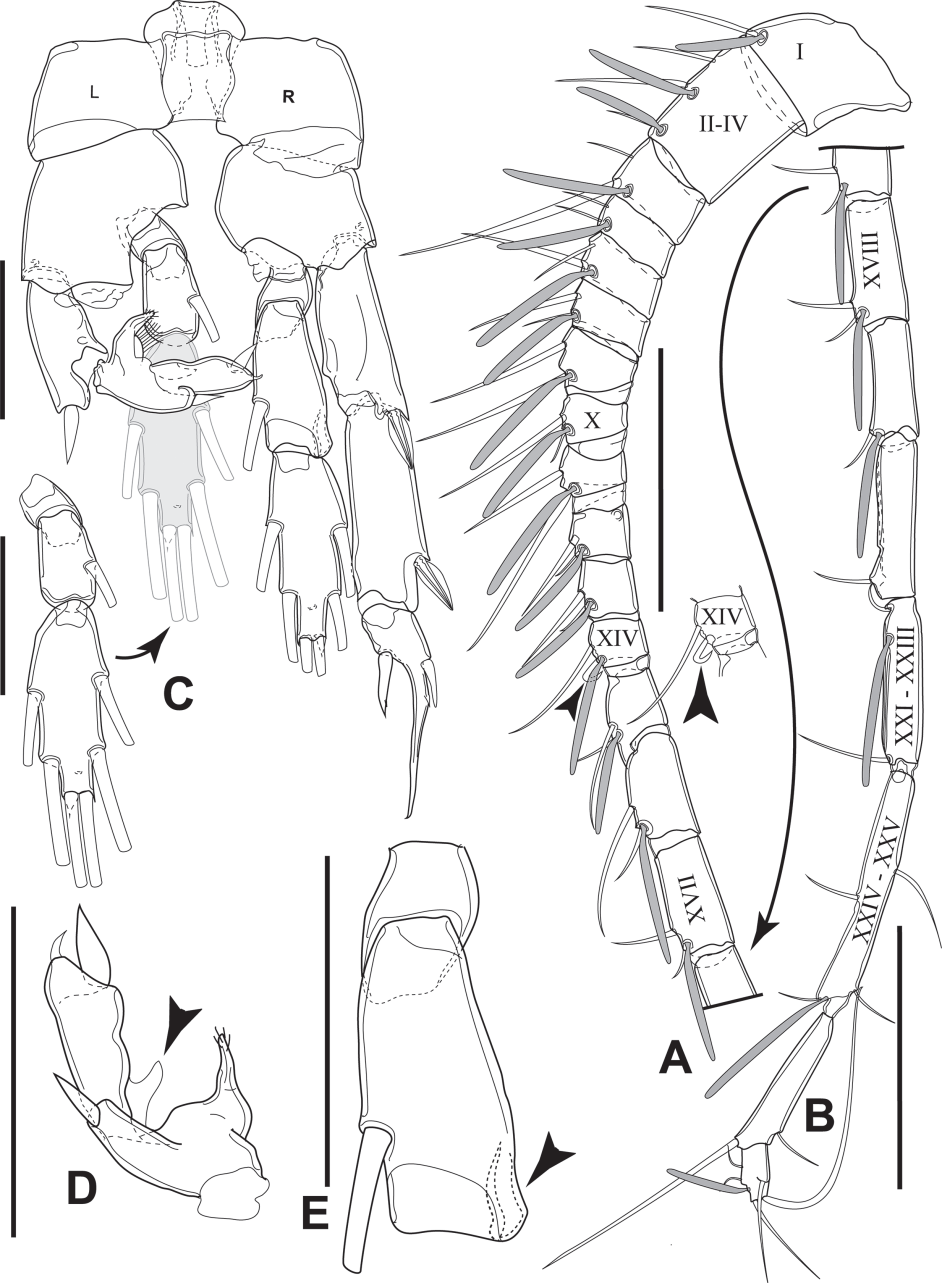
*Sipadantoniusroihani* gen. et sp. nov. male: **A** segments 1–15 of antennule, with picture of segment 12 that aesthetasc is removed **B** segments 16–22 of antennule **C** P5 furcal view **D**Exp-2 and Exp-3 of right leg of P5, lateral view **E**Endp-1 and Endp-2 of left leg of P5, posterior surface. Arrowheads indicate the important characteristic of the structure and Roman numerals on antennule correspond to ancestral segments. Scale bars: 200 μm (**A, B**); 50 μm (**C−E**).

Antennules asymmetrical. Left antennule non-geniculate, reaching distal margin of urosomite 2; articulation and setation identical to those of female. Right antennule geniculate, representing 22-segmented (Fig. 8A, B); ancestral segments II–IV completely fused, representing evident segment II; ancestral segment XIV with hook-like transformed seta; ancestral segments XXI–XXIII and XXIV–XXV completely fused representing evident segment XIX and XX, respectively; ultimate segments ~ 1/3 of the length of pre-ultimate segment; armature formula as follows (Roman numerals correspond to ancestral segment): 1+ae (I), 3+2ae (II–IV), 2+ae (V), 2 (VI), 2+ae (VII), 2+ae (VIII), 2+ae (IX), 2+ae (X), 2+ae (XI), 2+ae (XII), 2+ae (XIII); 1 hooked seta+1+ae (XIV), 2+ae (XV), 2+ae (XVI), 2+ae (XVII), 2+ae (XVIII), 1+ae (XIX), 1 (XX), 1+ae (XXI–XXIII), 4+ae (XXIV–XXV), 2 (XXVI), 5+ae (XXVII–XXVIII).

Antenna, mandible, maxillula, maxilla, maxilliped, and P1−P4 as those of female.

P5 (Fig. 8C−E) biramous, asymmetrical. intercoxal sclerite as described for female P3; coxa lacking medial seta. Left leg biramous; coxa and basis as shown; Exp and Endp three-segmented each. Exp-1 lacking inner seta, with conical, smooth outer spine; Exp-2 transformed, with claw-like extension on medial margin and conical, smooth outer spine; Exp-3 with conical, smooth subapical spine and minute apical spine, with claw-like cuticular expansion at base (Fig. 8C, D); Endp inserted on medial socket of basis; Endp-1 without inner seta, anterior surface longer than posterior one; Endp-2 shorter than that of right leg, with inner seta; Endp-3 with six marginal setae and cuticular pore on anterior surface. Right leg biramous; coxa and basis as shown; Exp and Endp three-segmented each; Exp-1 and Exp-2 elongate, each with one outer spine, but lacking inner seta each; Exp-3 with apical spine fused to segment bearing it, with spine on both lateral and medial margin; Endp-1 as that of right leg; Endp-2 longer than that of left leg, with longitudinal groove on distolateral corner and inner seta; Endp-3 as that of right leg.

##### Variability.

Based on three male specimens, including the allotype, the inner seta was absent from the right P4 Exp-1 in the allotype. There was no additional remarkable variation between the females and males.

##### Abnormality.

There was a curved suture on the right side of the ventral surface of the genital somite in one male (Fig. 7D).

##### Etymology.

The specific epithet was conferred in honour of Mr Roihan Han, a Malaysian deep dive record holder (at a depth of 164 m), who also led the Turtle Tomb exploration activity. Consequently, the name is a noun in the genitive singular.

##### Differential diagnosis and remarks.

The new taxon belonged to the superfamily Pseudocyclopoidea Giesbrecht, 1893, indicated by the following diagnostic characteristics mentioned in Bradford-Grieve et al. (2014):

1. fully developed the arthrodial membrane between body somites and limb segments,
2. identical configuration of mouthparts in both sexes,
3. segments I−VIII of Exp of the antenna with one seta each,
4. segment V of Endp of maxilliped with outer seta,
5. right antennule of the male geniculate,
6. P1–P5 with both three-segmented rami,
7. Exp-3 of P1–P5 with two, two, three, three, and three outer spines, respectively.

Epacteriscidae Fosshagen, 1973 and Pseudocyclopidae Giesbrecht, 1893 (Bradford-Grieve et al. 2014) are two families of primitive calanoid fauna that have been recently accepted within it. Among these two taxa, Epacteriscidae is the basal calanoid fauna from a phylogenetic perspective (Bradford-Grieve et al. 2010).

Following the recent morphological examination, the new species demonstrated a close affinity to the family Pseudocyclopidae through the presence of the following shared characteristics:

1. rostrum single plate with a rounded tip and without rostral filaments,
2. furcal ramus symmetrical and without modification of the left furcal seta VI,
3. mandibles with well-developed Endp forming the main axis of the mandibular palp,
4. maxillulae with marginal setae IX on coxal epipodite,
5. maxillipeds with normally developed endopod segments II−VI longer than coxa,
6. distolateral corner of Exp-2 the female P5 extended,
7. Exp-2 of the female P5 offset in oblique angle to the main axis of the rami.
8. Exp-2 of the left leg of the male P5 with the inner process.

It was noted that the new species exhibited an affinity to the family Epacteriscidae, characterised by the presence of aesthetasc on the ancestral segment IV of the antennule and the absence of inner process on Exp-2 of the right leg of the male P5. Furthermore, a combination of the presence of aesthetasc on the ancestral segment XIX of antennule in both sexes and the presence of aesthetasc on the ancestral segment XX of the female antennule, as identified in the new species, had been observed exclusively in *Caiconectes* Fosshagen & Iliffe, 2007. Nevertheless, the new species could not be classified as a member of this family due to its lack of a bilobed rostrum, rostral filament, and raptorial-adaptive features of mouthparts. An example of the last characteristic was the enlargement of the ventral teeth (e.g., Fosshagen et al. 2001; Fosshagen and Iliffe 2007). In light of this, the presence of the aesthetasc on the ancestral segment XX of the female antennule was probably convergent among the new species and *Caiconectes*. The taxonomic placement of the latter genus remained uncertain, having been more recently classified as incerta sedis. The assumption that *Caiconectes* belonged to the Epacteriscidae phylogenetic lineage, whereas the new species belonged to the Pseudocyclopidae lineage, was supported by the differences observed in the following characteristics between the new species and *Caiconectes*: configuration of the rostrum, feeding mode adaptive feature of mouthparts, ornamentation of the distolateral corner of the P1Exp-2.

Consequently, the placement of the new species within the superfamily Pseudocyclopoidea and the family Pseudocyclopidae was deemed justified. It was suggested that the presence of aesthetasc on ancestral segment IV of the female antennule and the absence of inner process on Exp-2 of the right leg of the male P5 constituted merely an occasional occurrence of the characteristic within the representatives of the justified family. Following the synonymisation of the families Boholinidae and Ridgewayiidae with the family Pseudocyclopidae, 14 calanoid genera were included. The genera *Boholina*, *Exumella*, *Placocalanus*, *Pseudocyclops* and *Ridgewayia* are polytypic; whereas the remaining genera are monotypic, represented by one species. Among these genera, the monotypic genus *Pinkertonius* is considered the basal taxon. The phylogenetic study indicated four characteristics that distinguished *Pinkertonius* from other genera: (1) the separation of ancestral segments II and III of the female antennule, (2) the presence of aesthetasc on the ancestral segment IV of the male antennule, (3) the presence of medial seta 4 on the female P5 Exp-3, and (4) the separation of Exp-2 and Exp-3 of both rami of the male P5. Based on the aforementioned characteristics, the new species represented one of the phylogenetic transitional stages between the genus *Pinkertonius* and other genera, as the same conditions of characteristics (2), (3), and (4) were present in the new species with separated ancestral segments II and III of the female antennule. Other characteristics shared by the new species and *Pinkertoniusambiguus* Bradford-Grieve, Boxshall & Branco-Bercial, 2014 included (1) the shape and the armament of Exp-3 and Endp of the right leg of the male P5, (2) the absence of armature element of maxillular basal exite, and (3) the relative length of ancestral segment XXVII of antennule.

Nevertheless, the new species was unable to be classified as a member of the genus *Pinkertonius* and all genera of the family Pseudocyclopidae based on the combination of the following characteristics:

1. furcal rami lacking seta III,
2. furcal seta VII inserted beside seta VI rather than in front of the seta V or seta VI,
3. antennule with aesthetasc on ancestral segment IV in both sexes,
4. ancestral segments XIX−XXIII of the male antennule without modified setae
5. maxillulae with six setae on coxal endite,
6. seta 4 of the endopodal segment II of maxilliped transformed,
7. basis of P1−P5 without lateral seta,
8. the male P5 Endp-1 lacking inner seta,
9. the right leg of the male P5 lacks process on the inner margin of Exp-2.

Consequently, the new generic rank *Sipadantonius* gen. nov. was established within the family Pseudocyclopidae, intended to accommodate the new calanoid species from the Turtle Tomb of Sipadan Island, Sabah, Malaysia. Furthermore, the female exhibited the most plesiomorphic characteristics of the family Pseudocyclopidae by having aesthetascs on the ancestral segments IV and XX of antennule and six setae on the maxillular coxal endite. The number of setae on the maxillular coxal endite in Calanoida exhibited variability; however, it was noted that there had never been more than five setae (Boxshall and Halsey 2004). To date, the presence of six elements on the coxal endite was recorded in the order Misophrioida Gurney, 1933, as well as the families Canuellidae Lang, 1944 and Longipediidae Boeck, 1865 (Boxshall and Halsey 2004).

## ﻿Discussion

The order Calanoida had been divided into nine superfamilies by Andronov (1974). Subsequently, after Fosshagen and Iliffe (1985) raised the family Platycopiidae Sars, 1911 to full order rank, namely Platycopioida Fosshagen, 1985 and following the introduction of two new superfamilies by Park (1986), ten superfamilies were recognised. They included Augaptiloidea Sars, 1905, Bathypontioidea Brodsky, 1950, Centropagoidea Giesbrecht, 1893, Clausocalanoidea Giesbrecht, 1893, Epacteriscoidea Fosshagen, 1973, Eucalanoidea Giesbrecht, 1893, Megacalanoidea Sewell, 1947, Pseudocyclopoidea Giesbrecht, 1893, Ryocalanoidea Andronov, 1974, and Spinocalanoidea Vervoort, 1951, in which Andronov (2007) suggested the merger of Epacteriscoidea within Pseudocyclopoidea. According to a morphology-based phylogenetic study, the monophyly of numerous recognised superfamilies was established (Bradford-Grieve et al. 2010); the finding was subsequently corroborated by the multiple gene-based phylogenetic analysis (Blanco-Bercial et al. 2011). Bradford-Grieve et al. (2014) indicated that the families Boholinidae, Pseudocyclopidae, Ridgewayiidae, and Epacteriscidae were included in the re-definition of the superfamily Pseudocyclopoidea, based on the recovery of the monophyletic relatedness among the superfamilies Epacteriscoidea and Pseudocyclopoidea. Although the clade was formed with low jackknife support due to a considerable number of homoplastic characters, evidence indicated that each superfamily possessed a distinct set of morphological characteristics that reflected the unique genetic basis of the superfamily. This may have been the reason that the previous gene-based phylogenetic analysis of the Calanoida aligned with the earlier morphology-based phylogenetic inference (Park 1986; Bradford-Grieve et al. 2010, 2014). In the case under consideration, it was observed that most of the ancestral characteristics of Pseudocyclopoidea were retained in the new species. They included a triangular rostral plate without a rostral filament, a complete separation of ancestral segment IV, which resulted in antennules with 27 segments, a maxilliped with six-segmented Endp, P5 with both rami three-segmented, separated genital apertures each hidden under its operculum, and presence of inner seta on Exp-1 of the female P5 (Andronov 2014). Furthermore, the weak expression of the sexual dimorphism of P5 and the configuration of swimming legs, which were regarded as indicative of the origin from a single species of the Pseudocyclopoidea (Andronov 2014), is relevant for the new species. The characteristic of P2 Exp-3 with two outer spines was observed in other families, including Calanidae Dana, 1846, Paracalanidae Giesbrecht, 1893, Pseudodiaptomidae Sars, 1902, Sulcanidae Nicholls, 1945, and Tortanidae Sars, 1902. The representatives of these families have P5 bearing Exp-3 with two outer spines at most. Furthermore, the considerable reduction of Enp of the female P5 and the strong expression of sexual dimorphism of P5 were evident in most of the aforementioned families (Boxshall and Halsey 2004; Andronov 2014).

Bradford-Grieve et al. (2014) noted certain modifications of the mandible, maxilla, and maxilliped that were associated with the families Epacteriscidae and Pseudocyclopidae, thereby indicating their feeding habits (e.g., Fosshagen 1968; Fosshagen et al. 2001; Jaume and Humphreys 2001; Fosshagen and Iliffe 2003, 2004a, 2004b, 2007; Suárez-Morales and Iliffe 2007; Boonyanusith et al. 2020). Recently, the family Epacteriscidae is comprised of *Balinella* Fosshagen, Boxshall & Iliffe, 2001, *Bofuriella* Fosshagen, Boxshall & Iliffe, 2001, *Bomburiella* Fosshagen, Boxshall & Iliffe, 2001, *Cryptonectes* Fosshagen & Iliffe, 2004, *Iboyella* Boxshall & Jaume, 2003, *Oinella* Fosshagen, Boxshall & Iliffe, 2001, *Bunderia* Jaume & Humphreys, 2001, *Edaxiella* Fosshagen, Boxshall & Iliffe, 2001, *Epacteriscus* Fosshagen, 1973, *Enantronoides* Fosshagen, Boxshall & Iliffe, 2001, *Enantiosis* Barr, 1984, *Enantronia* Fosshagen, Boxshall & Iliffe, 2001, *Gloinella* Fosshagen, Boxshall & Iliffe, 2001, *Minnonectes* Fosshagen & Iliffe, 2004, *Erebonectes* Fosshagen, in Fosshagen & Iliffe, 1985, and *Miheptneria* Fosshagen & Iliffe, 2004) had been considered as raptorial feeders, based on the characteristics such as the gnathobase of the mandible with enlarged ventral teeth, the mandibular palp with reduced Endp and the well-developed Exp forming the main axis of the palp, the transformation of setae on the distal part of the maxilla and maxilliped to stout, elongate, spinous setae or the reduction of setae on both appendages, and the maxilliped bearing a shortened Endp. On the other hand, the feeding habits of the family Pseudocyclopidae were observed to be more variable. Genera such as *Boholina*, *Hondurella*, *Ridgewayia* and *Stygoridgewayia* were presumably particle feeders (Fosshagen and Iliffe 2003; Suárez-Morales and Iliffe 2007; Tang et al. 2008; Boonyanusith et al. 2020), *Exumella* were considered to be a benthic scavenger, based on the reduction of the seta of the Endp of the mandible (Jaume and Boxshall 1995), in addition to *Exumellina* and *Stargatia* are believed raptorial feeders, deduced from the paddle-like adaptive feature of the mandible and the Endp of maxillule (Fosshagen and Iliffe 1998). The new species was presumed to be a particle feeder, demonstrating a close affinity with the family Pseudocyclopidae from a taxonomic perspective based on the four following morphological characteristics, which include: (1) the mandible armed with numerous small teeth on the cutting-edge of the gnathobase; (2) the well-developed Endp of the mandible with four and more than nine setae on the proximal and the distal segments, respectively, and representing the main axis of the mandibular palp; (3) the maxilla with elongate endite on the basis and armed with normal plumose setae; (4) the Endp of the maxilliped elongate. The new species exhibited a modification of a seta on the Endp of maxilliped, also observed in *Brattstromia* (Fosshagen and Iliffe 1991); however, the shape of the transformed seta differed between the new species and the genus mentioned above. In *Brattstromia*, the tip of the seta was serrated and sharpened, which related to its cutting function (Fosshagen and Iliffe 1991). In the new species, the tip of the seta was ornamented with long spinules, which bore a resemblance to the feather-like spine that has frequently been observed in the cave-dwelling harpacticoid genus *Elaphoidella* (Brancelj et al. 2010). It was hypothesised that the transformed seta on the maxilliped of the new taxon could be utilised as a collecting apparatus, in accordance with the assumption posited by Brancelj et al. (2010), who postulated that the modified seta plays a role in collecting food particles, such as fine detritus or bacteria.

The existence (= the presence/absence) of aesthetascs on certain segments of the antennule was employed in morphology-based phylogenetic analysis of the superfamily Pseudocyclopoidea (Bradford-Grieve et al. 2014). The presence of aesthetascs on all other ancestral segments was utilised for the analyses; I, II, III, VII, XVI, XXII, XXIII, XXIV, and XXVI–XXVIII of the ancestral segments were refrained. This was likely attributable to the low phylogenetic implication of the characteristic, which may have indicated the consistency in the existence of aesthetascs on the aforementioned ancestral structures in the superfamily Pseudocyclopoidea. *Pinkertonius* and *Sipadantonius* gen. nov., as well as the three basal taxa of the superfamily, namely *Caiconectes*, *Erebonectoides*, and *Azygonectes*, consistently exhibited a presence of aesthetascs on the ancestral segments I, III, VII, XVI, and XXVII–XXVIII and consistently lacking aesthetascs on the ancestral segments II, XXII, XXIII, XXIV, and XXVI (Fosshagen and Iliffe 1994, 2007; Bradford-Grieve et al. 2014). Thus, the presence or absence of aesthetascs in the segments mentioned above was presumably plesiomorphic in the superfamily. In contrast, the retention of aesthetascs on the ancestral segments IV, XIX and XX of the antennule in both sexes indicated that the new species also exhibited an affinity to the Epacteriscidae. Nevertheless, an inconsistency in the presence of an aesthetasc on a particular ancestral segment was frequently observed in the Epacteriscidae, a close relative of the Pseudocyclopidae, thereby suggesting that the characteristic was homoplastic. The initial instance of an inconsistent appearance in the retention of aesthetascs in the Epacteriscidae was observed in the genus *Oinella*; only the taxon lacked an aesthetasc on the ancestral segment IV of the female antennule (Fosshagen et al. 2001), whereas this characteristic was evident in females of all other genera. The retention of aesthetascs on ancestral segments XIX and XX was sporadically observed within the superfamily Pseudocyclopoidea, predominantly among the Epacteriscidae. In *Caiconectes*, an aesthetasc was present on the ancestral segments XIX and XX of the female antennules and on the segment XIX of the males (Fosshagen and Iliffe 2007). In the female of *Erebonectoides* and *Oinella*, as well as the male of *Enantiosis*, *Bomburiella* and *Exumella* (Fosshagen and Iliffe 1994; Jaume and Boxshall 1995; Fosshagen et al. 2001), an aesthetasc was documented to be present in the ancestral segment XIX of the antennule, and the retention of an aesthetasc on ancestral segment XX has been reported in the females of *Gloinella* (Fosshagen et al. 2001). The examples supported the assumption that the retention of aesthetascs in these three segments could occur independently within the superfamily Pseudocyclopoidea. Furthermore, the presence of a distolateral process on the P1Exp-2 and the absence of the rostral filament supported the placement of the new species within the family Pseudocyclopidae. Although the process was identified in several other genera, including *Badijella*, *Boholina*, *Stygoridgewayia*, *Pseudocyclops* and *Ridgewayia* (Ohtsuka et al. 1999, 2000; Kršinic 2005; Tang et al. 2008; Boonyanusith et al. 2020), it had not been reported in Epacteriscidae. In contrast, the rostral filament, which was consistently present in the representatives of Epacteriscidae, was absent in the new species. Moreover, the rostral filament has been reported from numerous genera of Pseudocyclopidae, such as *Badijella*, *Robpalmeria*, *Normancavia*, *Exumella* and *Pinkertonius*. The characteristics of their buccal appendages exhibited an adaptive feature of particle feeders or scavengers. Furthermore, the maxillular coxal epipodite which bore seta 9, as observed in the new species, differed from most of Epacteriscidae, in which seta 9 was absent (Jaume and Boxshall 1995; Fosshagen and Iliffe 2003; Kršinic 2005; Bradford-Grieve et al. 2014).

Five plesiomorphic characteristics of *Pinkertonius* were noted: (1) the female antennule segmentation between the ancestral segments II and III; (2) the male antennule with aesthetasc on the ancestral segment IV; (3) Endp-1 of the male right P5 with medial seta; (4) Exp-3 of the female P5 with medial seta 4; (5) the P5 with three-segmented Endp in both sexes. The new species exhibited a close phylogenetic relatedness with *Pinkertonius*, characterised by the presence of the combination of characteristics (2), (4), and (5), which served to distinguish the new species and *Pinkertonius* from all other Pseudocyclopidae. The characteristic (2) demonstrated a high phylogenetic signal in morphology-based phylogenetic analyses of the superfamily (Bradford-Grieve et al. 2014), having been shared by nearly all of the Epacteriscidae and three additional genera, namely *Azygonectes*, *Erebonectoides* and *Caiconectes*. The assumption regarding the close relationship between *Sipadantonius* gen. nov. and *Pinkertonius* was supported by the observation that the characteristic of the P5 with a three-segmented Endp in both sexes was shared by a few numbers of calanoid genera of Pseudocyclopidae which includes *Sipadantonius* gen. nov., *Pinkertonius* and *Exumella*. Nevertheless, the reduction of the Exp was deemed relevant in *Exumella* (Jaume and Boxshall 1995; Suárez-Morales and Iliffe 2005). Furthermore, the Endp of the male P5 was derived in various patterns of segmentation reduction in other Pseudocyclopidae. For instance, it was observed that the Endp of the male P5 exhibited a two-segmented ramus on one side and a three-segmented on the other one in *Brattstromia*, *Exumellina*, and *Stargatia* (Fosshagen and Iliffe 1985, 1998, 2003). In contrast, it bore a two-segmented ramus on both sides in *Badijella* and one-segmented rami on both sides in *Boholina*, *Hondurella*, *Stygoridgewayia* or *Robpalmeria* (Fosshagen and Iliffe 1989, 2003; Kršinic 2005; Suárez-Morales and Iliffe 2007; Tang et al. 2008). The sole significant distinction observed in the male P5 Endp between the new species and *Pinkertonius* was the reduction of the inner seta on the Endp-1.

Certain characteristics link *Pinkertonius* to the family Pseudocyclopidae, including: (1) the existence of an aesthetasc on a certain segment of the female antennule; (2) segmentation and armament of the Endp of the mandible; (3) the armament of epipodite of the maxillule; (4) ornamentation of the basis of the P1, and (5) segmentation and ornamentation of the male P5 (Bradford-Grieve et al. 2014). Based on the aforementioned characters, the new species and *Pinkertonius* were differentiated from one another by the characters (1), (2), and (5). Nevertheless, this study posited that the new species and *Pinkertonius* were close relatives due to their shared characteristics of having aesthetascs on the ancestral segment IV of the male antennule. Furthermore, the variation in the number of armature elements of Endp-2 of the mandible and ornamentation of Exp-2 of the male right P5 had commonly been observed in Pseudocyclopidae, even among the representatives of a monophyletic clade. This study, thus, posited that the differences in these two characters between the new species and *Pinkertonius* were merely the result of the reduction/transformation of the structure within the closely related phylogenetic lineage. The two examples that supported the argument were the differences in the number of the armature element of Endp-2 among the genera *Hondurella* and *Stygoridgewayia*, as well as the differences in the ornamentation with the medial process of Exp-2 of the male right P5 among the genera *Placocalanus* and *Pseudocyclops*. *Hondurella* and *Stygoridgewayia* were identified as the sister taxon, constituting a monophyletic clade in morphology-based phylogenetic analyses (Bradford-Grieve et al. 2014). However, *Hondurella* bore nine setae on Endp-2 of the mandible instead of 11 setae presented in *Stygoridgewayia* (Suárez-Morales and Iliffe 2007; Tang et al. 2008). Likewise, *Placocalanus* and *Pseudocyclops* constitute a monophyletic clade; however, the ornamentation with a medial process on Exp-2 of the male right P5 was present in *Pseudocyclops* but not in *Placocalanus* (Bradford-Grieve et al. 2014). The number of setae on Endp-2 of the mandible varied between six and eleven setae among the representatives of Pseudocyclopidae (Bradford-Grieve et al. 2014). Furthermore, the ornamentation with the medial process of Exp-2 of the male right P5 was absent in numerous genera, including *Boholina*, *Hondurella*, *Stygoridgewayia*, and *Placocalanus* (Ohtsuka et al. 1996; Suárez-Morales and Iliffe 2007; Tang et al. 2008; Boonyanusith et al. 2020).

It was noted that the new species bore six elements on the maxillular coxal endite and furcal rami, lacking furcal seta I and seta III. The presence of six elements on the maxillular coxal endite constituted the most plesiomorphic characteristic among the representatives of Calanoida, in which the maximum number of setae on the endite was five (Boxshall and Halsey 2004). On the other hand, the reduction of furcal seta I and sata III could be considered an apomorphic characteristic of the new species. These characteristics supported the placement of the new species outside either the family Pseudocyclopidae or the superfamily Pseudocyclopoidea. Nevertheless, the setation of the armament of the maxillular coxal endite and furcal rami exhibited variability, even within the same genus. The occurrence of six elements on the maxillular coxal endite was reported from the order Misophrioida, as well as the families Canuellidae Lang, 1944 and Longipediidae Boeck, 1865 (Boxshall and Halsey 2004). Examples of the difference in the setation of the maxillular coxal endite among representatives of a genus were *Misophriella* Boxshall, 1983 and *Longipedia* Claus, 1863. Six elements were present on the endite of both *M.schminkei* Martínez Arbizu & Jaume, 1999 and *L.ulleungensis* Bang, Moon & Back, 2021 (Martínez Arbizu and Jaume 1999; Bang et al. 2021). Furthermore, five elements were present in *M.tetraspina* Boxshall, 1983 and *L.koreana* Bang, Moon & Back, 2021 (Boxshall 1983; Bang et al. 2021).

A distinctive characteristic of the new species was the reduction of furcal setae I and III. In Pseudocyclopoidea, the complete reduction of furcal setae was reported for the furcal setae I and II, with the latter being specific to *Miheptneria* (Bradford-Grieve et al. 2014). In other families, the reduction of furcal setae was reported in *Temorites* Sars, 1900 of the Bathypontiidae Brodsky, 1950, and *Fosshagenia* Suárez-Morales & Iliffe, 1996, of the Fosshageniidae Suárez-Morales & Iliffe, 1996 (Sars 1900; Suárez-Morales and Iliffe 1996; Fosshagen and Iliffe 2004b). However, the retained setae were interpreted as seta III to seta VI (Bradford-Grieve et al. 2014). Thus, the reduction of furcal seta III was identified as a distinctive characteristic of the new species, thereby facilitating the classification of this new species within a newly established genus. An inconsistency in the setation of the furcal rami was found in certain other copepod taxa, even within the same genus, suggesting an influence of the ecological differentiation or the niche partitioning on the setation of furcal rami. An example was the genus *Pseudograeteriella* Brancelj, Boonyanusith & Sanoamuang, 2019 in which the variation of the armament of the furcal rami was found, even the two most closely related representatives were encountered within the same cave (Sanoamuang et al. 2019). Based on the evidence mentioned earlier, this study assumed that the presence of six elements on the maxillular coxal endite represented merely the retention of the plesiomorphic characteristic in the new species, while the reduction of the armament of furcal rami was influenced by the ecological differentiation, as had been observed in other Copepoda. Therefore, the new species could be classified in the family Pseudocyclopidae.

The superfamily Pseudocyclopoidea had frequently been reported from cave habitats, and it appeared likely that no relevant adaptive feature was specific to copepods inhabiting this type of environment. It was possible that such a morphological adaptation/modification was related to the zones of the water column in which the copepods lived (Boonyanusith et al. 2020). In general, the relatively long antennules (i.e., surpassing the prosome) were frequently observed in pelagic representatives collected from the water column of the cave, such as *Caiconectes*, *Azygonectes*, *Exumellina* and *Stargatia* (Fosshagen and Iliffe 1998, 2003, 2007). Conversely, the relatively short antennule, as found in some epacteriscids and pseudocyclopids like *Stygoridgewayia*, *Boholina*, and *Ridgewayia*, was deduced as a hyperbenthic adaptive feature (Fosshagen and Iliffe 1998; Tang et al. 2008; Boonyanusith et al. 2020). Other characteristics observed consistently in benthic copepods (Fosshagen 1973) included the compact body and strong spines on the exopods of the swimming legs (Bradford-Grieve 2002). Following the aforementioned assumption, the new taxon would be the pelagic adaptive Calanoida, characterised by long antennules surpassing the posterior margin of the second urosomite and conspicuous oar-shaped outer spines on the exopods of all swimming legs. In addition, it was posited that the hypothetical calanoid ancestor, as well as most of its relatives, are epi- or hyperbenthic colonisers (Huys and Boxshall 1991; Bradford-Grieve 2002). Thus, the colonisation of the pelagic realm was probably secondary, occurring subsequent to the divergence of the new species from the epibenthic ancestor.

The two assumptions regarding the pelagic adaptation and the secondary colonisation of the pelagic realm of the new species were likely interconnected with the geological history of the sampling area and the ecological characteristics of the cave. From a geological perspective, the Turtle Tomb is located beneath Sipadan Island, which is the top of a volcanic cone located within the complex zone formed by the convergence of three tectonic plates, namely the Philippines Sea, Indian and Pacific Plates (Bellon and Rangin 1991). Historically, the volcanic cone primarily originated from the vertical rising of the magma, followed by the extinction of the volcano and the subsequent growth of the corals on the extinct volcano. This indicated the richness of volcanic activities at the seabed in this area, attributable to the convergence of the Eurasian, Pacific and India-Australian Plates since the Miocene (Yang et al. 2024). The environmental catastrophes and toxic gases likely prompted an adaptation of the epibenthic ancestor of the new species for colonisation of the pelagic environment prior to the colonisation in the cave. Based on the richness of organic particles and the stability of the environmental conditions, the cave functions as a refugee for the new species.

The second possibility was hypothesised based on the ecological characteristics of the cave. The new species was collected in the Turtle Tomb, recognised as one of the largest marine caves of Southeast Asia, located at Sipadan Island, from ~ 20 meters below the sea surface within the cave. The cave has a large entrance measuring ~ 20 meters wide and a horizontal gallery with depths varying from ~ 18–21 meters. Crystal-clear water with settled sediment at the sampling site indicates a slow movement of water currents. The broad space of the cave, coupled with the slow movement of water currents, likely facilitates an adaptation towards the planktonic lifestyle, enabling organisms to navigate through the free water masses within the cave. *Exumellina* is another example of a copepod exhibiting a planktonic lifestyle, having been collected in the pelagic zone of a marine cave (i.e., Norman’s Pond Cave). *Exumellina* is characterised by a slender body and relatively small and equal outer spines of Exp of P1–P4 (Fosshagen and Iliffe 1998).The disjunct biogeographic distribution of cave-dwelling calanoids in the two extremities of the Tethys Sea has suggested the colonisation of the habitats where they inhibited prior to the closure of the Tethys Sea (Bradford-Grieve 2002; Belmonte 2022). In the family Pseudocyclopidae, five genera constitute a monophyletic clade, in which the genus *Ridgewayia* is regarded as the sister taxon of the other five genera, including *Boholina*, *Hondurella*, *Placocalanus*, *Pseudocyclops*, and *Stygoridgewayia* (Bradford-Grieve et al. 2014). Although *Ridgewayia* and *Pseudocyclops* are cosmopolites, the regional distribution of the other genera is relatively restricted, having been recorded from merely one or two geographical regions. In one extremity of the Tethys Sea, which corresponds to the western coast of the Pacific Ocean, the new species and the two genera of the aforementioned clade have been reported from Southeast Asia, including *Boholina* and *Pseudocyclops* (e.g., Fosshagen and Iliffe 1989; Ohtsuka et al. 1999; Boxshall and Jaume 2012; Moon and Soh 2014; Tran and Chang 2020). Additionally, *Stygoridgewayia*, *Placocalanus*, and *Ridgewayia* (two out of six species of the *typica* species-group, including *R.boxshalli* Barthélémy, Ohtsuka & Cuoc, 1998 and *R.flemingeri* Othman & Greenwood, 1988) have been reported along the western coast of the Pacific Ocean, (Ohtsuka et al. 1996, 2000; Tang et al. 2008), forming the Indo-West Pacific community. In the other extremity of the Sea, which corresponds to the coast of the Atlantic Ocean and the Mediterranean, *Placocalanus* and *Hondurella* have been found from the Honduran coast of the Caribbean Sea of the North Atlantic Ocean (Fosshagen 1970; Suárez-Morales and Iliffe 2007). *Placocalanus* is the only genus that has been found in the localities beyond the Caribbean Sea and the Mediterranean; moreover, the six other genera, namely *Exumella*, *Exumellina*, *Stargatia*, *Brattstromia*, *Normancavia*, and *Robpalmeria*, have been found only from these two regions (e.g., Fosshagen 1970; Grahame 1979; Fosshagen and Iliffe 1991, 1998, 2003; Jaume and Boxshall 1995; Suárez-Morales and Iliffe 2005). The disjunct pattern of the regional distribution previously described appeared to resemble that of the genus *Ridgewayia*, as most species of the *typica* species-group had been documented from localities beyond the Caribbean Seas and the Mediterranean. In contrast, nearly all the *gracilis* species and the *marki* species-groups had been found in the North Atlantic/Mediterranean (Ohtsuka et al. 2000). Ohtsuka et al. (2000) posited that the existence of a certain morphological species-group (= *marki* species-group) in both the Indo-West Pacific region and the North Atlantic/Mediterranean served to confirm the faunistic link between these two regions. Furthermore, the presence of *Hondurella* and *Placocalanus* constituted another example of the phenomenon mentioned above. Thus, the regional distribution of the family Pseudocyclopidae exhibited the Tethyan track.

## Supplementary Material

XML Treatment for Sipadantonius

XML Treatment for Sipadantonius roihani

## ﻿Additional information

### Author ORCIDs

Chaichat Boonyanusith https://orcid.org/0000-0003-1487-2160

Koraon Wongkamhaeng https://orcid.org/0000-0001-7671-8869

Abdul-Rahim Azman https://orcid.org/0000-0002-8964-6638

### Data availability

All of the data that support the findings of this study are available in the main text.
